# Categorization of Natural Whistled Vowels by Naïve Listeners of Different Language Background

**DOI:** 10.3389/fpsyg.2017.00025

**Published:** 2017-01-24

**Authors:** Julien Meyer, Laure Dentel, Fanny Meunier

**Affiliations:** ^1^Univ. Grenoble Alpes, CNRSGIPSA-Lab, Grenoble, France; ^2^Centre National de la Recherche Scientifique, Laboratoire sur le Langage, le Cerveau et la CognitionBron, France; ^3^The World Whistles Research AssociationParis, France; ^4^CNRS UMR7320, Laboratoire BasesCorpus, Langage, Nice, France

**Keywords:** vowel perception, whistled language, Spanish, perceptual integration, perceptual flexibility

## Abstract

Whistled speech in a non-tonal language consists of the natural emulation of vocalic and consonantal qualities in a simple modulated whistled signal. This special speech register represents a natural telecommunication system that enables high levels of sentence intelligibility by trained speakers and is not directly intelligible to naïve listeners. Yet, it is easily learned by speakers of the language that is being whistled, as attested by the current efforts of the revitalization of whistled Spanish in the Canary Islands. To better understand the relation between whistled and spoken speech perception, we look herein at how Spanish, French, and Standard Chinese native speakers, knowing nothing about whistled speech, categorized four Spanish whistled vowels. The results show that the listeners categorized differently depending on their native language. The Standard Chinese speakers demonstrated the worst performance on this task but were still able to associate a tonal whistle to vowel categories. Spanish speakers were the most accurate, and both Spanish and French participants were able to categorize the four vowels, although not as accurately as an expert whistler. These results attest that whistled speech can be used as a natural laboratory to test the perceptual processes of language.

## Introduction

Whistled speech is a natural speech register that enables distant communication by transposing spoken languages into whistles (for a review, see Meyer, 2015). Whistles have the advantage of traveling long distances with less degradation of the signal than spoken or shouted speech. During the process of adapting speech to whistling, the linguistic information is emulated, adjusted and concentrated into a phonetic whistle that remains intelligible to trained speakers but not to untrained ones, even if they fluently speak the language that is being whistled.

The strong reduction of the frequency spectrum that operates from ordinary modal speech to whistled speech is why this register requires additional training in both production and perception. The emulation at play results in a reduction/degradation of speech at the source made by the speakers themselves. This procedure is language specific, relying on the whistlers’ selection of salient features of a given language, which explains its major interest for phoneticians. To date, all over the world, approximately 40 low-density and remote populations are known to have adapted their local language to this special speech register (Meyer, 2015). Among these, different strategies of transposition from spoken speech to whistled speech have been observed depending on a major typological distinction: tonal languages, in which tones carried by the pitch of the spoken voice distinguish lexical or grammatical meanings, versus non-tonal languages.

In tonal languages (see Rialland, 2005 and Meyer, 2015, for reviews), in what is called ‘pitch-based whistling,’ whistles are focused on transposing the fundamental frequency (from the vibration of the vocal folds) to primarily encode the lexical tones. Therefore, in the whistled form of a tonal language, the vowel quality is completely excluded. The direct drawback is that the possibility to transmit complex sentences to a trained listener depends on the informational load carried by tonal prosody in this language: for example, it is much higher in the Hmong language (7–8 contour tones depending on the dialect) than in Surui (two level tones).

In non-tonal languages, in what is called ‘formant-based whistling,’ the strategy is different because whistlers carefully approximate the vocal tract articulation used in the spoken form. As a consequence, the pronunciation of whistled vowels and consonants is in direct relation to the specificities of the vocal tract maneuvres occurring in spoken speech (to the extent that they can be achieved while maintaining a whistle source in the anterior oral part of the mouth). This provokes a whistled adaptation of vowel and consonant qualities which are traditionally carried by the complex frequency spectrum of the voice (and partly by the main resonances of the sound in the mouth called formants by phoneticians). This means that whistlers traditionally learn to transform the complex perceptual attribute of timbre resulting from human voice into a simple modulated frequency line. For language scientists, it is a challenge to understand how this transformation works both in production and in perception. Indeed, strikingly, the whistled phonetic details that are selected during this procedure are sufficient for trained whistlers of non-tonal languages to recognize non-stereotyped sentences as well as to achieve a reasonable degree of word and syllable recognition. For example, in their seminal study on whistled Turkish, Busnel and colleagues showed that words are recognized at a rate of approximately 70%, whereas common whistled sentences are recognized at a rate of approximately 80–90% (see Busnel, 1970, Moles, 1970, and Meyer, 2015, for a review). It has been shown that systems of whistled vowels follow the same general organization in all the non-tonal languages. For example, in Greek, Spanish, and Turkish, whistled vowels are emitted at different pitch levels depending on the frequency distribution of their timbre in the spoken modal speech form (i.e., /i/ has a high pitch, /e/ lower, /a/ even lower, see Supplementary Figure 1 and see Meyer, 2008). In whistled speech, each individual whistled vowel is much more relative than in spoken modal speech because it depends solely on a simple frequency value approaching the characteristics of a simple sinewave. The identity of a given vocalic position (for example /e/ or /o/) can be ascertained only when confronted to other vocalic positions of the same language. Moreover, whistled frequencies can vary with the technique (bilabial whistle, finger whistle…) but also the physiology of the whistler and the power of whistling. For example, the farther the whistlers have to communicate, the higher is the whole scale of the vocalic frequencies, /i/ staying below 4 kHz and the lowest vowel above 1 kHz (Meyer, 2008). This is the reason why we generally study whistled productions of different whistlers separately, and even take care to group productions of a same whistler only if they were produced with the same whistling technique. For a given distance of communication, a given whistling technique and a given individual whistler, each vowel is whistled within a specific interval of frequency values covering the variability of articulation of the corresponding vowel position. Finally, the relation between whistling and its amplification in vocal tract resonances has been hardly studied. Yet, Shadle (1983) showed that a whistle frequency is always captured by either the second or third formant of the vocal tract – in a position for whistling – and that a frequency jump between the two occurs when these resonance areas – or formants – are close (which happens, for example, in front vowels).

Although whistled speech provides evidence that the tremendous capacity of the human brain is able to recognize speech from reduced acoustic cues and even from signals very different from voice, few studies have been conducted on whistled speech recognition or on whistled speech learning. One in neuroscience using fMRI showed that the brain areas traditionally associated with language are activated in well-trained listeners but not in untrained ones (Carreiras et al., 2005). Another study, using behavioral technique, showed that the traditionally reported left hemispheric lateralization of speech processing may be partially due to the acoustic properties of the signal, as whistlers showed more lateralization in syllable recognition when listening to spoken speech than when listening to whistled speech. Interestingly, whistled speech perception even challenges the traditional view in this domain as it results in a symmetric use of left and right hemispheres for simple syllable recognition (Güntürkün et al., 2015). A third recent study using behavioral measurement showed how naïve French listeners were able to categorize the whistled Spanish vowels /i, e, a, o/ quite similarly to a trained traditional Spanish whistler, even if the whistler does so more accurately (Meyer, 2008). This evidence demonstrated that the cognitive linguistic categorizations used to recognize spoken vowels are easily associated with tonal whistled frequencies by native speakers of a non-tonal language, without requiring any training. This result was an opportunity to explore why and how the quality of the spoken vowels can be adapted in a simple frequency for whistled speech and to understand more deeply the perception of vowels. As a consequence, taken together, such studies showed different fascinating possible directions of using the natural practice of whistled speech as a scientific object to examine various perceptual and neurocognitive aspects of speech.

In the present paper, we pursue this approach by extending the experiment of Meyer (2008) to naïve native speakers of the same language as the stimuli (Spanish speakers, as we again used the vowels /i/, /e/, /a/, /o/ of Spanish Silbo—the whistled version of Spanish of La Gomera Island) and naïve native speakers of a tonal language (Standard Chinese speakers). In tonal language such as Standard Chinese, changes in tone convey differences in the meaning and grammatical category of words. In our experiment as participants are naïve in whistled speech, their ability to categorize phonemes relies on perceptual flexibility, i.e., the ability to recognize a stimulus as belonging to a category even if slightly deviant from the acoustic form to which they are used. In the speech domain, perceptual flexibility corresponds to the ability to recognize words or other language units with novel pronunciations, such as those encountered in unfamiliar dialects and accents or in many different register of speech (Bent, 2015). This is an essential skill for children’s receptive language development for example, but also to learn a second language or understand someone who has a particular pronunciation. If participants of our studies categorize whistled vowels on the basis of their language specific system, we should observe differences in the pattern of categorisation depending of the language properties. In particular by testing Mandarin Standard Chinese participants, we wanted to see whether being native of a tonal language completely neutralizes the possibility to identify whistled vowels emulating spoken timbre. As a consequence we made the hypothesis that Spanish participants will be able to categorize whistled vowels much better than Standard Chinese participants even if both groups never heard any whistled speech stimuli before in their life.

We therefore looked at the different patterns of categorization for speakers with different native languages (Spanish, Standard Chinese, and French, reanalysing results previously obtained on French speakers). We also compared these patterns to a reference, i.e., the pattern observed for an expert whistler of La Gomera (reported in Meyer, 2008).

## Materials and Methods

In the experiments presented here, whistled Spanish vowels were presented on their own, without any context, first to native Spanish speakers (first experiment) and next to native Standard Chinese speakers (second experiment). The vowels selected for this study were the same as the stimuli used in Meyer’s (2008) experiment testing French speakers as participants in the same categorization task. Participants had to categorize whistled vowels in a simple and intuitive task.

### Participants

The participants were students at the University of Lyon (France) and teachers at the Instituto Cervantes of Lyon. The present experiments were conducted in accordance with the Declaration of Helsinki. The study was approved by the Comité d’évaluation éthique of SPIN-CNRS. All methods were carried out in accordance with the approved guidelines. All the participants provided informed consent. Participants filled a questionnaire asking which languages they had learned, whether they already knew about the existence of whistled speech and how long they had been learning French (with additional information about how long they had been living in France).

#### Spanish Listeners

Twenty volunteer native Spanish speakers (12 women and 8 men), living in Lyon and aged 19–34 years, took part in the experiment. None of the participants had known hearing loss. All of them had between 6 months and 9 years of experience learning French and therefore spoke enough French to understand the experiment instructions.

#### Standard Chinese Listeners

Twenty native speakers of Standard Chinese (17 women and 3 men), mostly students at the university Lyon 2 and aged 18–26, took part in the experiment. They had no known hearing deficit and spoke enough French to understand the experiment instructions. One participant was excluded from data analyses because he arrived in France at the age of 1 year old and spoke French all his life, while the others arrived as students and had between half a year and 8 years of experience learning French. None of them declared having learned Spanish.

### Stimuli

The sound extracts used as stimuli were selected in a corpus of Spanish whistled sentences recorded in 2003 by the first and second authors. The four tested vowels from the Spanish whistled language of La Gomera (Silbo) were: /i/, /e/, /a/, /o/. We did not include /u/ because in the spoken Spanish dialect of the island of La Gomera, on which is based the Silbo vocalic system, /u/ is rare (7%) and often pronounced as /o/ (Classe, 1957). Moreover, the expert Silbo whistlers with whom production studies were conducted were not found to whistle /o/ and /u/ at significantly different frequency levels (Classe, 1956; Rialland, 2005; Diaz Reyes, 2007; Meyer, 2008). Consequently, in whistled Spanish, the four vowels /i/, /e/, /a/ and /o, u/ show a frequency scale pattern with four significantly different intervals following a decreasing order of mean frequencies; this pattern holds across whistlers (e.g., Meyer, 2005). Importantly, /i, e, a, o/ vocalic realizations exist in the mother tongues of all participants tested here and in the previous study on French participants that we will analyze more deeply in the results. Indeed, in French, they exist as full vowel positions with pronunciations similar or close to Spanish (Calliope, 1989). In Standard Chinese, /i/ and /a/ correspond to full vowel positions, and /e/ and /o/ appear in some very specific contexts as variants of a mid vowel (Lee and Zee, 2003; Duanmu, 2007). Therefore, by testing Standard Chinese listeners on Spanish whistled vowels, we have the rare opportunity to observe various potential influences of a complex vocalic system in the perception of pure whistled pitch values: first, we may observe how Standard Chinese listeners categorize differently full vowel positions (/i/ and /a/) and variants of a same vowel (/e/ and /o/). But we will also be able to observe how they perceive these two categories in different conditions as /a/ and /e/ have two whistled frequency neighbors in whistled Spanish, whereas /i/and /o/ both have solely one whistled frequency neighbor in Silbo.

The experimental material consisted of 80 vowels (20 /i/, 20 /e/, 20 /a/ and 20 /o/), all extracted from the recording of 20 long semi-spontaneous sentences whistled relatively slowly in a single session by the same whistler in controlled conditions (same whistling technique during the entire session, constant distance from the recorder and from the interlocutor, and background noise between 30 and 40 dBA). In whistled speech, vowel nuclei are typically whistled as rather steady in frequency and are modulated at their extremity by the consonant articulation. Here, the sounds played as stimuli concerned only the vowel nucleus without the consonant modulations (see Supplementary Figure 1 where we present the spectrogram of 4 examples of stimuli presented to the participants, one for each vowel position).

**FIGURE 1:**
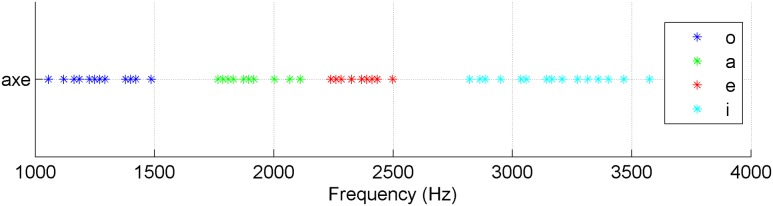
**Frequency distribution of played vowels of the experiments**.

They ranged from 1 to 3.7 kHz and were chosen in a confidence interval of 5% around the mean value of the frequencies of each vocalic interval, which means that, overall, the vowel frequency bands of the experiments do not overlap (**Figure 1**). The amplitudes of the stimuli were normalized to equal maximum values. Moreover, the durations at which the vowel nuclei were originally whistled in the sentences were kept for the stimuli. They ranged from 85 ms to 1 s with 71% of the vowels below 400 ms. The 29% remaining vowels corresponded to vowels lengthened by prosodic effects (as described in the literature on whistled Spanish, e.g., Classe, 1957; Meyer, 2008, and see Supplementary Table 1 in Supplementary Materials for the mean and standard deviation of each category as a function of the vowel type).

Altogether, our selected stimuli well represented the variability of pronunciation of the concerned whistled vowels in spontaneous whistled speech. Therefore, we remained in ecological conditions of natural productions and maintained enough variation to reveal finely the effects of frequency and duration in whistled vowel perception.

### Design and Procedure

The two experiments used the same design that included two phases: training and test. The training was composed of 40 whistle sounds (10 of each vowel, from four recordings of each vowel), presented in a fixed order. The test was composed of 64 whistle sounds randomly presented (16 of each vowel, from 16 different recordings per vowel). They were different from the sounds used in the training. Overall, each participant processed 104 stimuli.

The task was the same as that used by Meyer (2008): listening to a whistle sound followed by a four-alternative forced choice (4-AFC). The participants listened to a whistled vowel and immediately afterward selected the vowel type that he/she estimated was closest to the one heard by clicking on one of the four buttons corresponding to the French letters “a”, “é”, “i”, “o”.

The test was programmed in Flash-Actionscript and presented to participants on a computer in a quiet room using high-quality Sennheiser headphones (HD 449). To start, the participant listened to one whistled Silbo sentence to discover the type of stimuli he/she would have to process. This allowed the participant to check the volume comfort, approximately 70 dB. Then, the training started, and the participant listened to each stimulus one by one and categorized them as “a”, “é”, “i”, or “o” on the interface in French by clicking on the display (written “a”, “e”, “i”, “o” for convenience in the rest of the paper). Clicking started the next trial. The test followed directly. Only one listening was possible per stimulus, and there was no feedback. Overall, the experiment lasted 20 min. The time taken to answer was not recorded, but only the answer itself (“a”, “e”, “i”, or “o”).

## Results

**FIGURE 2:**
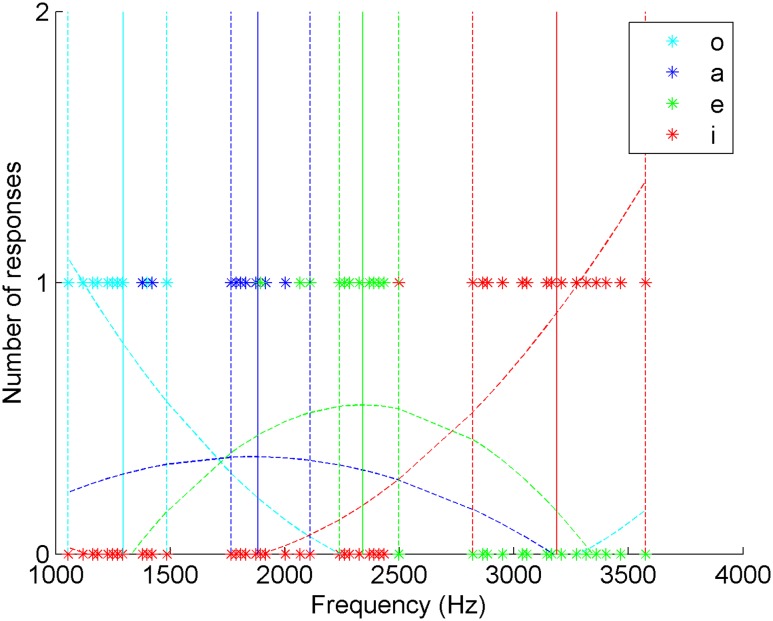
**Distribution of the answers of a native and fluent La Gomera whistler of whistled Spanish and estimated curves of whistler’s answers as a function of the frequencies of the Spanish whistled vowels**. For each vowel the plain vertical line represents the mean frequency value of the played vowels and the dotted lines represent the extreme frequency values of the corresponding interval.

**FIGURE 3:**
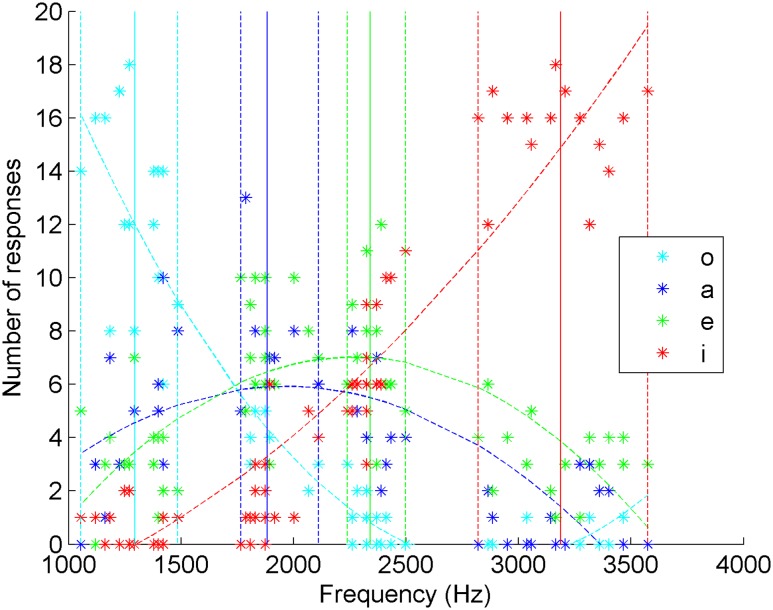
**Distribution of the answers of 20 Spanish non-whistlers as a function of the frequencies of the Spanish whistled vowels**.

**FIGURE 4:**
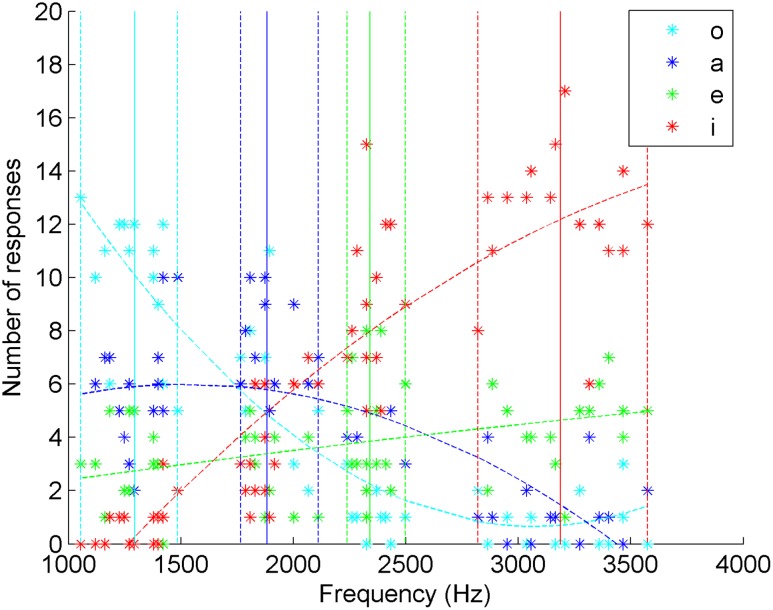
**Distribution of the answers of 20 Mandarin Chinese naïve listeners as a function of the frequencies of the Spanish whistled vowels**.

First and for reference for the present protocol, we present the whistled vowel identification pattern for a single native whistler of La Gomera, as observed previously in Meyer (2008). Next, we present the data for Standard Chinese and Spanish participants. Moreover confusion matrices of the answers (“i”, “e”, “a”, “o”) as a function of the played vowels (/i/, /e/, /a/, /o/) were derived from the participants’ answers (see **Table 1B** for all Spanish participants and **Table 1C** for all Standard Standard Chinese participants). Two different types of analyses were performed: first, concerning the pattern of correct answers; and next, the intervocalic confusions. Correct answers and confusions were also presented together by visualizing the answers of the participants as a function of the acoustic frequency of the whistle of each played vowel (like in **Figure 2**, see **Figure 3** for Spanish and **Figure 4** for Standard Chinese). This additional approach had the advantage of reintegrating in the analysis some information regarding the acoustics of the stimuli to enable precise determination of its influence on the patterns of answers. We first present these results separately for each participant population (Spanish, Standard Chinese) and finally compare the behaviors of the two groups with the group of French speakers as found in Meyer (2008; to which we apply the same procedure of analysis as developed for Standard Chinese and Spanish). A general ANOVA analyses including Language (Standard Chinese, Spanish, and French) and Vowel type (/a/, /e/, /i/, /u/) as main factors showed an effect of Language [*F*(2,56) = 6,139; *p* = 0.039], an effect of Vowel [*F*(3,168) = 47,153; *p* < 0.001] and crucially a significant interaction between the two factors [*F*(6,168) = 2,335; *p* = 0.0342], revealing that identification of the different vowels depended on participant native language. Therefore we analyzed the results for the different groups independently.

**Table 1:** Confusion matrix for the answers (in %) of an expert native whistler **(A)**, of 20 untrained Spanish native speakers **(B)**, of 20 untrained Standard Chinese native speakers **(C)**, and of 20 untrained French native speakers **(D)**.

		Answered vowels			
		≪ o ≫	≪ a ≫	≪ e ≫	≪ i ≫
**(A) Whistler**
Played	/o/	*87.50*	**12.50**	0	0
Vowels	/a/	**6,25**	*75*	**18.75**	0
	/e/	0	**6.25**	*87.50*	**6.25**
	/i/	0	0	**0**	*100*
**(B) Spanish**
Played	/o/	*62,5*	**20,63**	14,06	2,81
Vowels	/a/	**18,13**	*35,31*	**36,87**	9,69
	/e/	4,69	**25,31**	*35,31*	**34,69**
	/i/	1,56	4,38	**16,25**	*77,81*
**(C) Standard Chinese**
Played	/o/	*52.63*	**29.60**	14.14	3.62
Vowels	/a/	**28.60**	*35.53*	**16.77**	19.07
	/e/	8.88	**23,02**	*21,71*	**46.38**
	/i/	5.26	6.25	**24.34**	*64.14*
**(D) French**
Played	/o/	*50.63*	**40.31**	7.5	1.56
Vowels	/a/	**13.44**	*44.06*	**31.56**	10.94
	/e/	5.94	**22.19**	*46.88*	**25**
	/i/	0	4.38	**17.19**	*78.44*

*Values in italics correspond to correct answers and values in bold correspond to confusions with neighbouring-frequency vowels)*.

We focus here on the influence of the frequency value of whistled vowels on the answers, as it was the only varying parameter of the stimuli that was found to affect them. Testing for duration effect of the stimuli we contrasted ‘prosodically lengthened vowels’ and ‘non-prosodically lengthened vowel’ –as described previously in the introduction- and no significant difference was found in the type of correct answers “o”, “a”, “e”, “i” neither for Spanish [*F*(1,57) = 0.97, *p* = 0.325], nor for Standard Chinese participants [*F*(1,57) = 0, *p* = 0.975]. As we did not find any influence of duration on the answer pattern of the naïve participants in our experiments we did not integrate this factor further in our analyses.

### Reference Spanish Whistler of La Gomera

**Table 1A** show the performance on whistled vowel categorization by a single native whistler of La Gomera. He reached 87.5% of correct answers, confirming that a native whistler practicing nearly daily Spanish whistled speech identifies very precisely the four whistled vowels. The vowels were categorized significantly differently [X2(9) = 125.77, *p* < 0.001]. The agreement of the answers with the vowel categories was different from chance – which is at 25% in the present forced-choice protocol - and not accidental, as it was ‘substantial’ according to Cohen’s Kappa statistics (*k* = 0.79, *z* = 10.94, *p* < 0.001), which give a quantitative measure of the magnitude of such an agreement while being adjusted for agreement because of random chance alone (Cohen, 1960; Bishop et al., 1975).

The distribution of the confusion matrix showed that /i/ is perfectly identified and that the most difficult vowel to recognize for this whistler was /a/, which still reached correct identification of 75%.

To provide a more detailed view of the perceptual results, the collected data are also presented in **Figure 2** with the details of the influence of the frequency of the played vowels on the answers. In this figure, the estimated curves of the answers appear averaged by polynomial interpolations of the second order. **Figure 2** shows that the maxima of the estimated curves of whistler’s answers are always within 5% of variance of the range of variation of the vowels and therefore within the range of stimuli distribution for each vowel position. This graphical representation shows that a traditional whistled speech practitioner is very accurate in identifying whistled vowels of his own language.

### Spanish Naïve Listeners

In **Table 1B**, we present the confusion matrix of the Spanish listener. Considering the protocol and the task with four possible answers, these results show a different categorization for the four vowels [X2(9) = 833.02, *p* < 0.001]. The agreement of the answers with the vowel categories was again different from chance and not accidental, as it was almost ‘moderate’ according to Cohen’s kappa (k) statistics (*k* = 0.37, *z* = 22.62, *p* < 0.001).

#### Correct Answers

The mean level of success corresponding to correct answers was 53%. An analysis of variance (ANOVA) was performed on correct responses with ‘Vowel played’ (/a/, /e/, /i/, /o/) as a within factor. The scores varied significantly depending on vowels [*F*(3,57) = 22.42, *p* = 1.2e-9]. /a/ and /e/ gave the lowest scores (35.1%), while /o/ and /i/ were better recognized, with 62.5 and 78% of correct categorizations, respectively. *Post hoc* multiple *t*-tests with Bonferroni correction (*p* < 0.05) confirmed that /a/ and /e/ are less well recognized than /i/ and /o/, with no difference within each vowel group. These lower performances for /a/ and /e/ can be partly explained by the fact that they both have two perceptual neighbors in terms of pitch, a situation which multiplies the possibilities of confusion in comparison to the more isolated vowels /i/ and /o/.

#### Confusions

Observation of the confusions allowed us to better understand the results. First we looked at confusions between vowel types. We ran multiple *t*-tests with Bonferroni correction (*p* < 0.05) on all answers to explore vowel effects on confusion patterns. The vowel /o/, which represents the extreme category of lower whistled frequencies, is significantly different from its unique direct whistled vowel neighbor /a/, despite the levels of confusion on both sides of the matrix (/o/ is answered “a” in 20.63% of the cases confusing /o/, and /a/ is answered “o” in 18.13% of the cases confusing /a/). By contrast, intermediate vowels /a/ and /e/ are largely taken for one another to the point of not being significantly different in both directions (/a/ answered “e” and /e/ answered “a”). Moreover, confusions between /e/ and /i/ show an asymmetry depending on the vowel that is played: /i/ is answered “e” in 16.25%, but “e” and “i” answers remain significantly different when /i/ is played, whereas /e/ is much more often answered “i” (34.69%), and the test shows no significant difference between “e” and “i” answers when /e/ is played. Finally, it appears that the extreme vowels (/i/ and /o/) follow an effect of distance: the more the frequencies of the vowels are different, the less the vowels are confused. For example, for /o/, the confusion rates are as follows: “a” (20.63%) > “e” (14.06%) > “i” (2.81%; **Figure 3**).

Next, such as for **Figure 2** which dealt with answers of an expert Spanish whistler, the answers were presented as a function of the frequency distribution of the whistled vowels. They show that the maximum of estimated curves is always within 5% variance of the range of the vowels’ variation and therefore within the range of stimuli distribution. For “a” answers, the maximum of the curve estimating the number of answers was attained at a frequency of 1965.9 Hz which is within the frequency range of /a/ played vowels (from 1765.7 to 2110.3 Hz). For “e” answers, it was attained at a frequency of 2268.5 Hz which is within the frequency range of /e/ played vowels (from 2239.5 to 2497.9 Hz). Moreover, the estimated curves of answers “i” and “o” for extreme frequency vowels /i/ and /o/ have concave estimated curves reflecting lower degrees of confusions with neighboring vowels than correct answers. Together, these criteria show that the listeners categorize the vowels accurately in accordance with the vowel production, even for /a/ and /e/ despite their high inter confusions.

### Standard Chinese Naïve Listeners

The second experiment was exactly the same as the previous one, with the only difference being the population tested: native Standard Chinese speakers. The same analysis procedure applied to the Spanish listeners was used.

The four vowels were categorized differently [X2(9) = 565.65, *p* < 0.001], and the agreement of the answers with the vowel categories was not accidental (Cohen’s kappa *k* = 0.25, *z* = 114.58, low ‘fair agreement,’ *p* < 0.001).

#### Correct Answers

The mean level of success corresponding to correct answers was 43.5%. The scores varied significantly depending on vowels [*F*(3,54) = 17.20, *p* = 5.19e-8]. /e/ gave the worst performances (21.7%), below chance, while /a/ reached 35.5% of correct categorization, /o/ 52.6% and /i/ 64.1%. Finally, *post hoc* multiple *t*-tests with Bonferroni correction (*p* < 0.05) showed that /a/ and /e/ are significantly less well categorized than /i/ and /o/, with no significant difference within each vowel group in this test.

#### Confusions

The distribution of answers in a typical vocalic confusion matrix (**Table 1C**) shows different behaviors from the one observed for the Spanish. First, even if the whistled expression of the vowel position /i/ is the best recognized, confusions show that the categorization of /i/ and /e/ is not so clear because /e/ is highly mistaken for “i”, to the point that it is answered “i” twice as often as “e”. Bonferroni multiple *t*-tests (*p* < 0.05) were run on all answers. /e/ is often answered “a”, and this type of confusion is not significantly different from the correctly answered “e”, clearly showing that /e/ is also not well differentiated from /a/. Moreover, the “o” answers for /a/ were not significantly different from the group of correctly answered “a”. However, in the opposite direction of frequency values – /o/ answered “a” and /a/ answered “e” – the confusion rates result in a good discrimination of neighboring vowels, as they were significantly smaller than rates of correct answers. Again, there is an effect of distance only for the vowels situated at the extreme (/i/ and /o/). For example, for /o/, the confusions follow the order: “a” (29.38%) > “e” (13.44%) > “i” (3.45%).

To better understand the general pattern of confusions and correct answers, we also looked at the distribution of answers as a function of the played frequencies of each played vocalic item (**Figure 4**). Here, the tendency curves of “a” and “e” have their maximum outside of the frequency range of these vowels, which contrasts with what was obtained for Spanish listeners. Indeed, for “a” answers, the maximum of the curve estimating the number of answers was attained at a frequency of 1528.7 Hz which is below the minimum frequency of /a/ played vowels (1765.7 Hz). However, /a/ is much better recognized than /e/. The low and rectilinear tendency curve of “e” reflects that it has been very poorly recognized and has been much more often answered “i” and even more often answered “a” than “e”. The fact that it is also often mistaken for “o” explains that the tendency curve of “e” answers approaches a flat shape. Moreover, the tendency curve of “i” is convex, reflecting that /e/ as well as the highest frequencies of /a/ were both mistaken for “i” at higher rates than correct answers. All these aspects therefore show very high levels of confusion between vowels, indicating that Standard Chinese listeners have perceived the whistled vowel space very differently from the Spanish ones. Only /o/ was very well recognized along this representation.

Despite the lesser results of the Standard Chinese listeners, these listeners were good at relating high pitch whistles with vowels with rather acute timbres in the spoken form (/i/, /e/) and low pitch whistles with vowels with rather low timbres (/a/, /o/). Indeed, we found a different categorization of these enlarged categories [X2(1) = 323.106, *p* < 0.001)] and 75% of correct answers in the corresponding collapsed matrix.

### French Listeners, a Reanalysis

For a more complete picture of the influence of different native languages on the confusion patterns of whistled vowels by naive listeners, we extended the same procedure as for Standard Chinese and Spanish participants to the analysis of answers made by native French participants (data previously collected with French speakers in a test by Meyer (2008). It must be noted here that in the questionnaires filled by the French listeners – to which we had access - none of them declared having learned Spanish).

The results of **Table 1D** show a different categorization for the four vowels [X2(9) = 900.37, *p* < 0.001]. The agreement of the answers with the vowel categories was different from chance and not accidental, as it was ‘moderate’ according to Cohen’s Kappa (*k* = 0.4, *z* = 24.42, *p* < 0.001).

#### Correct Answers

The mean level of success corresponding to correct answers was 55%. The scores varied significantly by vowel (cf. **Table 1D**) [*F*(3,57) = 12.36, *p* = 2.46e-6]. Again, /a/ and /e/ gave the lowest scores (44.1 and 46.9%, respectively), while /o/ and /i/ were best recognized, with 50.6 and 78.4% of correct categorizations, respectively. *Post hoc* multiple *t*-tests with Bonferroni correction (*p* < 0.05) showed that /a/, /e/, /o/ are less well recognized than /i/, with no difference in the group of the three vowels.

#### Confusions

**FIGURE 5:**
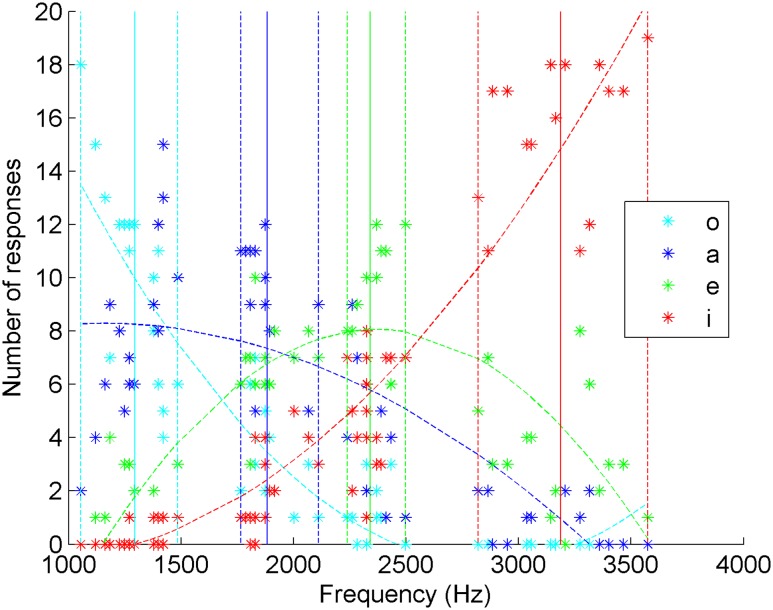
**Distribution of the answers of 20 French non-whistlers as a function of the frequencies of the Spanish whistled vowels**.

Like for the Spanish and Standard Chinese listeners, vowels were generally confused with their neighboring-frequency vowels (83% of the cases of confusion: bold letters in **Table 1D**). Performance variability also depended on vowel: /i/, the best recognized, had a correct answer rate that was significantly different from the others (multiple *t*-tests with Bonferroni correction, *p* < 0.05 on all answers). At the other extreme of whistled frequencies, French participants mistook /o/ for “a”, but not /a/ for “o”—no significant difference between “o” and “a” answers when /o/ was played. In contrast to Spanish and Standard Chinese participants, /a/ was the least well-identified, often miscategorized as “e” (no significant difference only in this direction of confusion). To better understand the general pattern of correct answers and confusions, we looked at the distribution of answers as a function of the played frequencies of each played vocalic item (**Figure 5**). It shows that French participants successfully categorized the three vowels /i/, /o/ and /e/ but not /a/. Indeed, for “a” answers, the maximum of the curve estimating the number of answers was attained at a frequency of 1153.4 Hz which is below the minimum frequency of /a/ played vowels (1765.7 Hz) and even within the frequency range of /o/ played vowels (from 1155.1 to 1485.8 Hz). As for the estimated curves of answers “i” and “o” for extreme frequency vowels /i/ and /o/, we found that they have concave estimated curves reflecting lower degrees of confusions with neighboring vowels than correct answers.

### Comparison between Language Groups

In the preceding sections, we developed different analyses of the perceptual behaviour of listeners by dealing successively with: (i) correct answers, (ii) rates of confusions between vowels, and (iii) answer distributions as a function of the frequency values of the stimuli (natural whistled Spanish vowels). These different approaches provide different insights into the effect of the language groups of listeners. Note that it was important to take into account the influence of the frequencies of the played whistled vowels on the answers of the participants (as proposed in iii) because of the highly relative nature of whistled vowels which are fully characterized acoustically by one simple frequency level.

**FIGURE 6:**
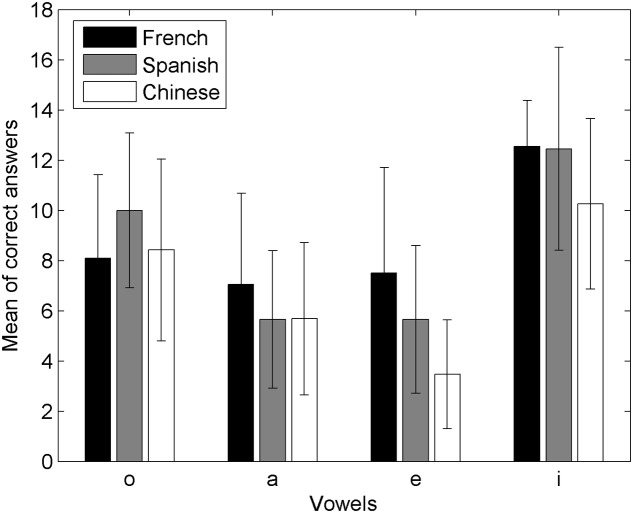
**Correct answers on each vowel for French, Spanish, and Mandarin Chinese native speakers**.

To compare the rates of correct answers among the three populations of naïve listeners (**Figure 6**), we ran additional comparative analyses: multiple *t*-tests with Bonferroni correction (*p* < 0.05) on the whole data, with the language Group as a between factor and focusing on effect of the Group. We found that the French and Spanish listeners’ scores were not significantly different, but that they were significantly better than the Standard Chinese listeners’.

As we have discussed in the preceding sections, analyses of the answers as a function of the distribution of the whistled vowels enable us to find further details. Indeed, they show that naïve Spanish listeners behave the most similarly to the expert whistler and are also the most faithful to the distribution of played vowels (accurate categorization of /i/, /e/, /a/, /o/), followed by the French (accurate categorization of only /o/, /e/, /i/) and last by the Standard Chinese native speakers (accurate categorization of solely /o/).

**FIGURE 7:**
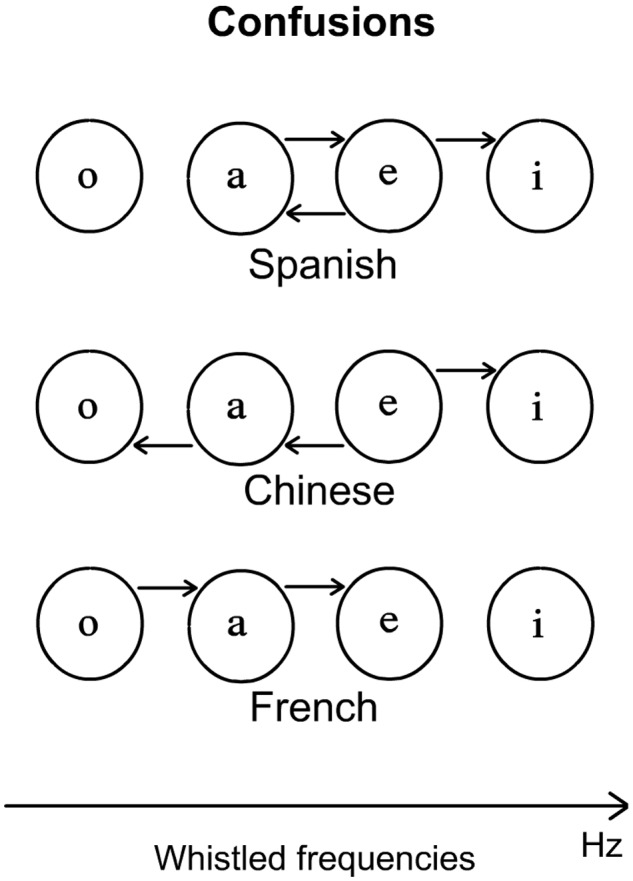
**Arrows show the confusions made depending on the native language**. For example, for Spanish listeners the arrow form /e/ to /i/ means that /o/ is taken for an /i/.

Finally, confusion rates in the three populations of naïve listeners showed some common tendencies, as most of the confusions were logical in the sense that a vowel was generally confused with neighboring-frequency vowels. However, we also found that participants’ confusions differed depending on their native language (see a graphical summary on **Figure 7**). Typically, naïve Spanish participants highly mistook /a/ and /e/ Spanish whistled vowels and also significantly mistook /e/ for /i/. Standard Chinese listeners contrasted largely with the Spanish, as they tended to mistake /e/ for both “a” and “i” and also /a/ for “o”. Finally, French participants also behaved differently from the two other groups of participants, as they mistook /o/ and /a/ with the vowel position corresponding to their higher neighbor in terms of interval of pitch frequency (**Figure 7**). An additional interesting comparison can be made: globally, when summing the confusions and the correct answers per answered vowels (columns in the confusion matrices of **Table 1**), we find that the whistler and the Spanish naïve listeners tend to answer more “e” and “i” whereas French and Standard Chinese naïve listeners tend to answer more “a” and “i”. The reasons of these general effects are explained by the different confusions we already detailed and will be discussed in the next section but we may already note that, in this respect, Spanish naïve listeners are again more faithful to the behavior of the expert whistler than the other two language groups.

## Discussion and Conclusion

To better understand the relation between whistled and spoken speech perception, in this paper, we explored how listeners of different native languages who were naïve in whistled speech categorized four Spanish whistled vowels. We ran experiments with Spanish and Standard Chinese native speakers and compared their results to those of French listeners and an expert Spanish whistler.

The fact that the two populations of Spanish and French naïve listeners managed to categorize the whistled vowels /o, a, e, i/ showed that they rather easily associated tonal whistled stimuli emulating vocalic quality to cognitive linguistic categorizations used for spoken vowel recognition. The differing results between Spanish, French and Standard Chinese listeners are instructive. Even if the French participants were the best in general performance (55%; just above Spanish, 53%, and largely above Standard Chinese, 45%), we found that they were neither significantly better than the Spanish in correct answers nor the most accurate when taking into account the distribution of answers as a function of the acoustic frequency of the whistle. Indeed, Spanish listeners were found to categorize the four whistled vowels most accurately, as their answers matched both the production values and the pattern of answers of the expert whistler, whereas the French accurately categorized only three vowels (o, e, i), and the Standard Chinese accurately categorized solely one vowel (o). This means that the Spanish, French, and Standard Chinese participants categorized the whistled vowels in three different ways, confirming that the listeners did not perform a purely acoustic task but were influenced by linguistic considerations related to the vocalic space of their mother tongue.

The fact that the listeners from the language of the stimuli were the most accurate when taking into account the distribution of answers as a function of the acoustic frequency of the whistle shows that Spanish whistled speech carefully emulates the spoken Spanish vocalic space in accordance with its spoken specificities.

By contrast, the Standard Chinese native speakers did not manage to categorize the four vowels well. Crucially, we found that a main perturbation was the low identification of /e/ whistled vowels. The absence of a clear phonologic [e] position in Standard Chinese (Duanmu, 2007) may explain this effect. Indeed, the great proportion of /e/ mistaken for “i” is probably because [e] appears in Standard Chinese only in two contexts: syllable-final preceded by a palatal glide or before [i]. Such co-occurrences that imply direct coarticulations between /e/ and /i/ in Standard Chinese contribute to the Standard Chinese participants’ association of high frequency values of /e/ whistled stimuli with [i] position. Interestingly, in Standard Chinese, [o] is an allophone of [e] and does not have a clear phonologic position either. [o] appears only associated with 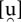 in Standard Chinese, but this could not affect the answers “o” because [u] was not tested in our study. So, confusions on /e/ and good categorization of /o/ might for a great part be due to the presence of /i/ and the absence of /u/ in the test. Interestingly, the Standard Chinese listeners’ little experience with the French language was apparently not of great help for the identification of /e/, which shows that they still used the vocalic system of their mother tongue as reference during this experiment, and not the one of their second language (L2). Therefore, the Standard Chinese listeners did not have enough experience in French to switch to the vocalic system of the French listeners.

Another important result of our experiment was that Standard Chinese listeners were able, to some extent, to associate tonal pitches with the quality of the spoken vowels. Indeed, they were good at relating high pitch whistles with vowel qualities characterized by rather acute timbres (/i/, /e/) and low pitch whistles with vowel qualities characterized by rather low timbres (/a/, /o/). Strikingly, this happened in spite of the fact that pitches in spoken Standard Chinese already express phonologic oppositions of tones. (The Standard Chinese listeners are used to performing a direct association between a pure pitch and the phonological tone carried by the vowel nucleus.) This means that having a tonal language as mother tongue is not fatal to the perception of vocalic qualities as pitch. Of course, the participants selected here had learned a non-tonal language (French); therefore, to conclude definitely on this point, a replication of this experiment would be necessary with Chinese listeners who have never learned a non-tonal language. If such further studies find that listeners are completely prevented by their tonal system in recognizing whistled vowels, this would mean that the Standard Chinese participants of the present experiment managed to neutralize their sensitivity to tone thanks to their experience with French. However, if results similar to ours are found, this would mean that Standard Chinese always manage to associate pitch to timbre.

The results also typically show that Spanish and French listeners were able to associate tonal pitches with the quality of the spoken vowels /i, e, a, o/ and with much more detail than for Standard Chinese. This shows that the frequency distribution of whistled vowels in a frequency scale – with /i/ identified as an acute vowel, /o/ as a low vowel, and /e/ and /a/ in between (/e/ a little higher in pitch than /a/) –is perceptually relevant to non-whistlers of these two languages. (by extension, the results suggest that a frequency scale may play an important role in the process of identification of the spoken Spanish and French vowels /i, e, a, o/). This ability must be rooted in underlying perceptual processes at play in spoken speech. Interestingly, several previous vowel perception experiments found a distribution of vowels in frequency scales. Most notably, these were based either on the notion of perceptual integration between close formants (Chistovitch and Lublinskaya, 1979) or on the notion of an effective upper formant (F2’) (Carlson et al., 1970; Bladon and Fant, 1978). These notions highlight that some aspects of the auditory system undergo a qualitative change when the spacing between two spectral prominences (such as formants) becomes less than a critical value of 3.5 bark (Stevens, 1998). The proximity of acoustically compact areas of the frequency spectrum (such as formants) favors their integration into a unique perceptual entity, showing a perceptual merging phenomena at the frequency level. This phenomenon was called the “Center of Gravity” effect and was originally found on vowel spectra (CG or CoG) (Chistovitch and Lublinskaya, 1979). It has been shown to play an important role in vowel identification because greater formant convergence explains better performance in vowel identification and a better stability of vowels in short-term memory (e.g., Schwartz and Escudier, 1989). Moreover, this phenomenon also explains why vowels of different spectra can be matched with the same vowel (Fox et al., 2011). The results of the French speakers could be analyzed under this frame. Indeed, they show good identification rates of whistled Spanish vowels but also confusion types that are more inaccurate than Spanish listeners’. This could be explained by the fact that the French vocalic system shares the four vocalic positions of the test with the Spanish vocalic system, but they are distributed differently in a larger vocalic inventory (Calliope, 1989; Meunier et al., 2003; Elvin et al., 2014; Kartushina and Frauenfelder, 2014). /i/ is a very extreme vowel in French because of its boundary with /y/, which pushes its phonetic characteristics to high formant values (e.g., Schwartz and Escudier, 1989; Rochet, 1991). In other words, the fact that the Spanish highly mistook /e/ for “i” while it was less mistaken in French might be caused by the fact that the focus of /i/ in Spanish is best defined by a focus on F2–F3 proximities, while it is rather characterized by a focus on F3–F4 for the French /i/. In parallel, the strong confusion of the whistled Spanish /a/ answered “o” by French participants is coherent with the differences between vocalic spaces of both languages, as the French [a] is more dissimilar from the French [o] than from the Spanish [o] in both F2 and F1, which are close formants in both vowels.

Finally, testing participants of different language backgrounds with whistled vowels enabled us to further reflect on the perceptual nature of pitch and timbre in voice and on the importance of perceptual integration processes in vowel quality perception and categorization. For formant-based whistling, whistled speech produces a different perception of a fully intelligible sentence, despite the elimination of canonical acoustic correlates of phonemes from the spectrum. Such a dramatic change in perception could be interpreted as an example of “perceptual insight” or pop-out and of a top–down perceptual process produced by higher-level knowledge and expectations concerning sounds that can potentially be heard as speech (much like what happens in Sine Wave Speech, see Davis and Johnsrude, 2007, but with a more drastic reduction to only one sine wave). A portion of this perceptual flexibility (Bent, 2015) is illustrated by the results of our study on vowels. Indeed, we have been able to observe the notion of perceptual flexibility at two levels. First, listeners were able to categorize vowels from non-standard but natural whistled vocalic articulations. Next, their native language background influenced them differently in perceiving whistled Spanish vowels. Moreover, our results on vowel confusions are coherent with the literature on the perceptual merging of close enough spectral prominences (CoG effect on vowel spectra) in the auditory-to-phonetic projection, which is interesting as this function is critical for increasing the perceptual salience of phonemes and for establishing the perceptual integrity of sound streams in modal spoken speech. Generally speaking, such developments in the interpretation of the results mark the high potential of whistled speech in serving as a tool to investigate perceptual processes in languages.

## Author Contributions

JM proposed the project. JM, FM, and LD designed and developed the experimental protocol and the set-up. JM and LD recorded whistled Spanish sentences in La Gomera. JM selected and edited the stimuli used as broadcast sounds in the experiment. FM facilitated approval to conduct the experiment at the Laboratory L2C2 of Bron. FM, JM, and LD performed the analysis.

## Conflict of Interest Statement

The reviewer MB declared a shared affiliation, though no other collaboration, with the authors JM and FM to the handling Editor, who ensured that the process nevertheless met the standards of a fair and objective review. The other author declares that the research was conducted in the absence of any commercial or financial relationships that could be construed as a potential conflict of interest.
