# Supplementary material for: Categorization of Natural Whistled Vowels by Naïve Listeners of Different Language Background
Source: Front Psychol. 2017 Jan 24;8:25. doi: 10.3389/fpsyg.2017.00025 (PMC5258750; doi:10.3389/fpsyg.2017.00025)
Supplement: Supplementary file 1 [file Data_Sheet_1.pdf]

frontiers

Supplementary Material

Categorization of natural whistled vowels by naïve listeners of different language background

Julien Meyer*, Laure Dentel, Fanny Meunier

* **Correspondence:** Corresponding Author: julien.meyer@gipsa-lab.fr

1 Supplementary Table 1: Mean and standard deviation values for 'prosodically lengthened' (t<400 ms) and 'non prosodically lengthened'(t>400 ms) categories as a function of each vowel type.

	Mean (t<400 ms)	Std (t<400 ms)	Mean (t>400 ms)	Std (t>400 ms)
o	171.3	64.4	648.8	161.2
a	237.7	41.4	675.6	180.3
e	203.8	67.4	702	237.9
i	176.5	53.1	508.6	59.7

2. Supplementary Figure 1:

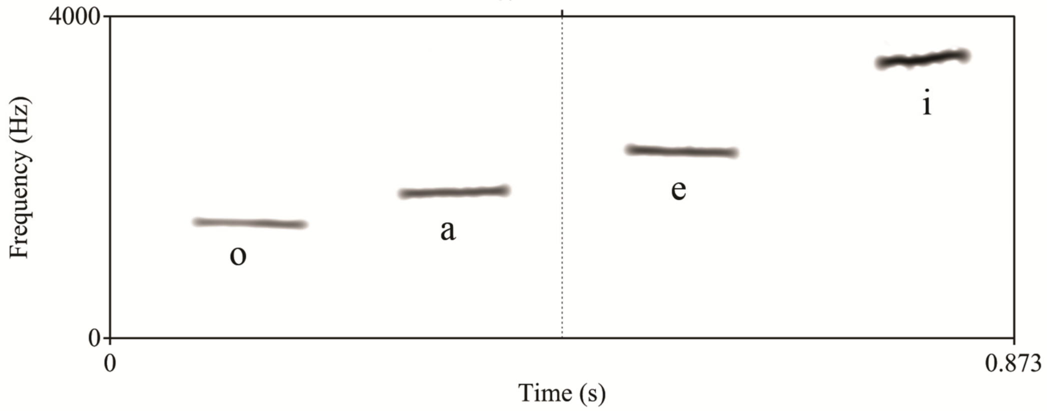

Supplementary Figure 1. Spectrogram of 4 examples of stimuli presented to the participants, one for each vowel type (/o/, /a/, /e/, /i/). This illustrates the typical frequency scale of whistled Spanish vowels.
